# In silico multi-epitope Bunyumwera virus vaccine to target virus nucleocapsid N protein

**DOI:** 10.1186/s43141-022-00355-y

**Published:** 2022-06-20

**Authors:** Kanaka Durga Devi Nelluri, Manne Anupama Ammulu, M. Lakshmi Durga, Melika Sravani, Vemuri Praveen Kumar, Sudhakar Poda

**Affiliations:** 1 Department of Pharmaceutics and Biotechnology, KVSR Siddhartha College of Pharmaceutical Sciences, Vijayawada, Andhra Pradesh India; 2 Department of Civil Engineering, PVP Siddhartha Institute of Technology, Kanuru, Vijayawada, Andhra Pradesh India; 3 Department of Biotechnology, Koneru Lakshmaiah University, Vaddeswaram, Andhra Pradesh India; 4 Department of Biotechnology, Acharya Nagarjuna University, nagarjuna nagar, Guntur, Andhra Pradesh India

**Keywords:** Bunyumwera virus, Multi-epitope, Nucleocaspid N-Protein, Vaccine design

## Abstract

**Background:**

Bunyumwera virus can cause 82% mortality in humans currently with no vaccine or drugs for treatment. We described an in silico multi-epitope vaccine targeting Bunyumwera virus nucleocapsid N-protein and predicted B and T cell epitopes for immunogenicity, allergenicity, toxicity, and conservancy. For creating the most potent immunological response possible, docking epitopes with HLA alleles are chosen to screen them. The 3D vaccination was docked with the Toll-like receptor-8 using molecular dynamic simulations. To ensure production efficiency, the vaccine sequence was further cloned in silico in a plasmid pIB2 vector. For efficacy and safety, results must be supported in vitro and in vivo.

**Results:**

The vaccine was cloned to enable expression and translation in a plasmid vector pIB2. It was expected to be antigenic, non-allergenic, and have a high binding affinity with TLR-8 in silico cloning. This multi-epitope vaccination may stimulate both innate and adaptive immunity.

**Conclusion:**

The vaccine developed in this work was based on the nucleocapsid N-protein of the Bunyumwera virus and was created using a reverse vaccinology method. Further experimental validation is required to assess the vaccine’s therapeutic effectiveness and immunogenicity.

## Background

In the *Bunyaviridae* family, the *Bunyamwera* group is one of 18 serologically discovered arbovirus serogroups in the *Orthobunyavirus* genus. They are made up of three single-stranded RNA segments along with nucleoproteins. *Bunyamwera* virus is prevalent in sub-Saharan Africa and is a leading cause of severe fever sickness in humans. The virus was identified from people in Uganda, Nigeria, and South Africa, and antibodies have been discovered in humans throughout sub-Saharan Africa, with a high frequency (up to 82%) in some places [1]. The virus was isolated from multiple *Aedes* species mosquitos, indicating that they are the primary carrier. Cache Valley Fever virus was recently characterized as a *Bunyamwera* virus strain, extending the infection’s total geographic distribution to North America. Other *Bunyamwera* virus strains were discovered in Argentina. In humans and mammals, the *Bumyamwera* virus-related illness was found to induce minor symptoms such as fever, joint discomfort, and rash. The Bunyamwera virus family consists of 32 viruses; among them, the viruses have the primary host as human – Batai, Bunyamwera, Fort, Germiston, Guaroa *Ilesha*, *Ngari*, *Shokwe*, and *Xingu* [2].

Bumyaviruses have a nucleocapsid protein (NP) that aids in the encapsidation of genomic RNA and viral replication. In the form of ribonucleoprotein complexes, copies of the N protein encapsulated genomic RNA segments. The N protein is employed in many serological and molecular diagnostics because it is the most abundant in viral particles and infected cells [3]. Using silico techniques, this work aimed to create an effective epitope-based peptide vaccination based on the known nucleocapsid N protein sequence of *Bunyamwera* virus.

## Methods

### Secondary structure analysis and recovery of the target protein's amino acid sequence

The amino acid sequence of the virus’s nucleocapsid protein N was obtained in FASTA format from the National Centre for Biotechnology Information (NCBI) database accession number AKX73307.1. At a threshold of 0.4, the online server VaxiJen v2.0 [4] predicted the antigenicity of the target protein (http://www.ddg-pharmfac.net/vaxijen/VaxiJen/VaxiJen.html). ProtParam [5] calculated the estimated instability index half-life, grand average, and aliphatic index of hydropathicity of the target protein (GRAVY-grand average of hydropathicity) (https://web.expasy.org/protparam/). The conformational sheet, helix, coil, and turn predicted by SOPMA used to identify the secondary structure of the viral structural protein N (https://npsa-prabi.ibcp.fr/cgi-bin/npsa_automat.pl?page=/NPSA/npsa_sopma.html).

### B cell and T cell epitope prediction

BepiPred-2.0 linear epitope prediction Immune-Epitope Database and Analysis-Resource (IEDB)^3^ used to predict B cell lymphocyte epitopes from target protein sequences. Eight epitopes with peptide lengths of more than nine mer were chosen for further investigation from the predicted epitopes (https://www.iedb.org/). The NetCTL 1.2 service [6] was used to predict MHC class-I restricted CD8+ CTL epitopes in the target protein sequence. For the 12 most commonly occurring HLA class I alleles in humans, such as A1, A2, A3, A24, A26, B7, B8, B27, B39, B44, B58, and B62, the NetCTL 1.2 service predicts 9-mer CTL epitopes (http://www.cbs.dtu.dk/services/NetCTL/). The weights on C-terminal cleavage and TAP transport efficiency were 0.15 and 0.05, respectively, during the CTL Epitopes prediction, while the threshold value for Epitopes identification was 0.75 [7]. The NetMHCIIpan 3.2 service was used to predict MHC class-II restricted CD4+ HTL epitopes. For HLA Class II DRB1 alleles, 15-mer HTL epitopes were found in the following sequences: 01:01, 03:01, 04:01, 07:01, 08:03, 10:01, 11:01, 12:01, 13:02, 14:01, and 15:01, with strong and weak binder thresholds of 2 and 10%, respectively. These HLA class II alleles were chosen because they encompass 95% of the global population [8].

### Prediction of epitope properties

The antigenicity of both B cell and T cell epitopes was predicted using VaxiJen v2.0 with a threshold of 0.4 (http://www.ddg-pharmfac.net/vaxijen/VaxiJen/VaxiJen.html). Both B cell and T cell epitopes were tested using AllerTOP v2.0 [9] to determine their allergenicity (https://www.ddg-pharmfac.net/AllerTOP/). ToxinPred8 was utilized to predict the toxicity of both B and T cell epitopes using a 10-amino-acid peptide fragment (http://crdd.osdd.net/raghava/toxinpred/). Epitope antigens that were non-allergic or toxic were utilized.

### Epitope conservancy and population coverage

To assess diversity and degree of conservancy in protein sequences from multiple countries, the IEDB conservation-analysis-tool [10] was used to check epitope linear sequence conservancy of projected B and T cell epitopes. The epitopes that were found to be 100% conserved were further investigated (http://tools.iedb.org/conservancy/). Utilizing the default factors, sequences of predicted CTL epitopes and their restricted MHC alleles were succumbed to the IEDB population coverage analysis programme (http://tools.iedb.org/population/).

### 3D structure modeling and molecular docking

RPBS MOBYL portal’s online PEPFOLD 3 server 10 was used to construct the de novo 3 dimensional structures of the chosen T cell epitope sequences (https://bioserv.rpbs.univ-paris-diderot.fr/services/PEP-FOLD3/). In PDB format, five models of each peptide sequence were generated. HLA-A*01:01 (HLA class I allele) and HLA-DRB1* 15:01 (HLA class II allele) X-ray diffraction structures with PDB ID 4U6Y and 1BX2 were acquired from Protein Data Bank (PDB). The Computed Atlas of Surface Topography of Proteins (CASTp) [11] software was used to find functional pockets in receptors (http://sts.bioe.uic.edu/castp/calculation.html). The docking analysis was carried out in PyRxVina, and multiple ligand-protein docking was done, in which this software gives binding affinity results for every ligand, PyMOL version 1.7.4.4 (Schrodinger). The postures of docked complexes were visualized using a molecular graphics technology.

### Vaccine sequence construction

Finally, vaccinations were developed using selected epitope sequences. BCL epitopes were developed after adjuvant, HTL epitopes, and CTL epitopes. The L7/L12 ribosomal protein was used as an adjuvant in the vaccine design to improve vaccination immunogenicity. AAY, EAAAK, KK, and GPGPG linkers were used to link the adjuvant and selected epitopes for vaccine manufacturing. The adjuvant sequence is linked by the EAAAK linker, whereas the CTL, HTL, and BCL epitopes are linked by the AAY, GPGPG, and KK linkers, respectively.

### Prediction of various vaccine properties

VaxiJen v2.0, AllerTop v.2.0, ToxinPred, and ProtPram tools were used to predict the final vaccine design’s allergenicity, antigenicity, toxicity, and other physicochemical characteristics.

### Structureal modeling, modification, and confirmation of vaccine

The secondary structure of the final vaccine built was predicted using the SOPMA [12] secondary structure prediction method tool by setting the output width, similarity threshold, and window width to 70, 8, and 17, respectively (https://npsa-prabi.ibcp.fr/cgi-bin/npsa_automat.pl?page=/NPSA/npsa_sopma.html). A 3D structure modeling was done in an online server PHYRE 2[13] protein fold recognition serve (http://www.sbg.bio.ic.ac.uk/phyre2/). Following that, more finer was used to improve the 3D vaccination model created (https://zhanggroup.org/ModRefiner/). Saves v6.0 was used to validate the revised 3D vaccination model. Six different programmes are used by the SAVES metaserver to validate the submitted protein structure (https://saves.mbi.ucla.edu/). ERRAT [14], Verify3D [15], WHATCHECK, and also analyzed Ramachandran Plot [16] by using PROCHECK.

### Molecular docking of vaccine with the receptor

Toll-like receptor-8 (TLR-8) is thought to have a role in the immune response to RNA viruses, according to several studies [17]. As a result, the vaccine 3D structure’s was docked against TLR-8. TLR-8’s X-ray diffraction structure (PDB ID: 3W3G) was acquired from Protein Data Bank with a resolution of 2.3 A0 (PDB). HawkDock was used to do the docking analysis (http://cadd.zju.edu.cn/hawkdock/).

### Molecular dynamic simulation of the docked complex

On an Internet server called Anisotropic network model web server 2.1, a molecular dynamics simulation of a receptor vaccination complex was performed (http://anm.csb.pitt.edu/) [18]. Molecular dynamic simulations were used to evaluate the receptor-vaccine complex interaction’s stability and investigate the physical mobility of atoms and macromolecules.

## Results

### Structural analysis of target proteins

The structural nucleocapsid protein N sequence of the Bunyamwera virus was 233 amino acids long, according to NCBI. The target protein had an antigenicity score of 0.5713, a molecular weight of 26621.75 kDa, and an isoelectric point of 9.30, with 25 negatively charged and 31 positively charged residues. The average hydropathicity was −0.216, with an instability index of 28.22, and aliphatic index of 87.42, and an average instability index of 28.22. The secondary structure prediction indicated a 41.63% alpha-helix, 20.17% extended strands, 5.15% beta-turn, and 33.05% random coil shape.

### B cell epitope prediction

In this research, we developed 32 epitopes, eight of which were tested for peptide length >9 and 100% conservation in viruses sequenced in different countries, as shown in Table 1. Out of 8 epitopes, only two antigenic, non-allergenic, and non-toxic properties offering epitomes were selected, of which SGLGWKKTNVSA showed maximum antigenicity (1.8250).

**Table 1:** B cell epitope antigenicity, allergenicity, toxicity, and topology

S. No	Peptide sequence	Antigenicity	Allergenicity	Toxicity	Topology
1	EFNDVAANTSSTFDPEV	Antigen (0.8395)	Allergen	Non-toxic	Outside
2	FKRIYTTGLSY	Non-antigen (0.1418)	Allergen	Non-toxic	Inside
3	GREIKTSLTKRSEWE	Antigen (0.7975)	Allergen	Non-toxic	Inside
4	TNFPGNRNSPVPDDGL	Non-antigen (−0.4624)	Non-allergen	Non-toxic	Outside
5	KVSEPEKLI	Non-antigen (−0.2748)	Non-allergen	Non-toxic	Inside
6	PLAEKNGITWSDG	Non-antigen (0.1285)	Non-allergen	Non-toxic	Outside
7	RKEMEPKYLEKTMRQRYMGLEASTW	Antigen (0.8312)	Non-allergen	Non-toxic	Inside
8	SGLGWKKTNVSA	Antigen (1.8250)	Non-allergen	Non-toxic	Inside

### T cell epitope prediction

With 261 peptides of 9-mer length, we identified 20 HLA class I supertypes. We chose 12 peptides with 100% conservancy and affinity for various HLA Class I alleles (Table 2). KRSEWEVTL (1.5287) had the highest antigenicity, while HTL epitopes did not overlap with HLA epitopes. And the 4 peptides, which were antigenic, non-allergic, and non-toxic were chosen for further population coverage study.

**Table 2:** MHC Class-I epitopes antigenicity, allergenicity, toxicity, and topology

Position	HLA class 1 alleles & supertypes	Peptide sequence	Antigenicity	Allergenicity	Toxicity	Topology
9	A_1,_A_24,_B_7_,B_8_,B_39_,B_58_,B_62_	VAANTSSTF	**Antigen** **0.4577**	Allergen	Non-toxic	Inside
15	A_1_,A_3_,A_26_,B_8_,B_27_,B_39_,B_58_,B_62_	STFDPEVAY	**Antigen** **0.7863**	Allergen	Non-toxic	Outside
69	A_1_,A_3_,A_24_,A_26_,B_7_,B_8_,B_27_,B_58_,B_62_	KVTVFNTNF	**Antigen** **0.5884**	Non-allergen	Non-toxic	Inside
149	A_1_,A_3_,A_24_,A_26_,B_8_,B_27_,B_39_,B_58_,B_62_	EMFLGTFKF	**Non-antigen** **0.0983**	Non-allergen	Non-toxic	Outside
197	A_1_,A_2_,A_3_,A_26_,B_7_,B_8_,B_39_,B_58_,B_62_	KVNEVQSAL	**Non-antigen** **0.3378**	Allergen	Non-toxic	Inside
143	A_1_,A_24_,A_26_,B_8_,B_39_,B_44_,B_58_,B_62_	SFFPGSEMF	**Non-antigen** **−0.0452**	Non-allergen	Non-toxic	Outside
28	A_1_,A_3_,A_26_,B_7_,B_8_,B_27_,B_58_,B_62_	RIYTTGLSY	**Antigen** **0.4063**	Allergen	Non-toxic	Inside
94	A_1_,A_2_,A_3_,A_26_,B_8_,B_27_,B_58_,B_62_	RLSGFLARY	**Non-antigen** **0.3265**	Non-allergen	Non-toxic	Outside
221	A_1_,A_26_,B_7_,B_8_,B_27_,B_58_,B_62_	AAREFLAKF	**Non-antigen** −**1.2944**	Allergen	Non-toxic	Inside
118	A_1,_B_7,_B_8_,B_39,_B_44,_B_62_	IKSKIINPL	**Antigen** **0.4284**	Non-allergen	Non-toxic	Inside
55	A_1,_B_8,_B_27,_B_39_	KRSEWEVTL	**Antigen** **1.5287**	Non-allergen	Non-toxic	Inside
57	A_1,_A_2,_B_7,_B_8,_B_27,_B_39,_B_44_	SEWEVTLNL	**Antigen** **0.7108**	Non-allergen	Non-toxic	Outside

**Fig. 1:**
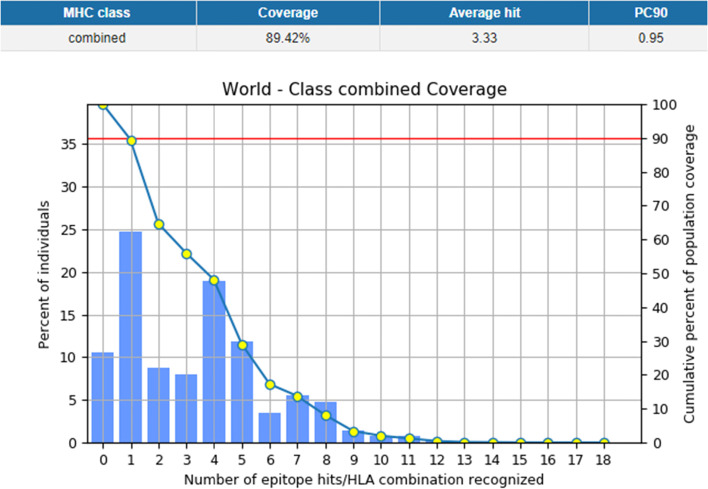
MHC class 1 epitopes coverage worldwide

With 89.42% global coverage (Fig. 1), 4 CTL epitopes were submitted to population coverage analysis in IEDB against their restricted MHC alleles. Europe (96.21%) had the largest population coverage, followed by North America (88.61%) and East Asia (86.88%). For the anticipated epitopes, the cumulative percentage of population coverage was calculated. The results are shown in (Table 3).

**Table 3:** Population coverage by selected MHC class-I epitopes

Population	Coverage
World	89.42%
East Asia	86.88%
North East Asia	65.36%
South East Asia	72.63%
South West Asia	73.06%
Europe	96.21%
East Africa	74.33%
West Africa	68.23%
Central Africa	63.13%
North Africa	72.24%
South Africa	53.38%
North America	88.61%

In the NetMHCIIpan 3.2 server, 65 binding solid peptides were identified as possible HTL epitopes, of which 12 bound firmly to multiple HLA class II alleles with 100% conservancy (Table 4) and three were chosen for vaccine development.

**Table 4:** MHC Class-II epitope antigenicity, allergenicity, toxicity, and topology parameters

Position		Peptide sequence	Antigenicity	Allergenicity	Toxicity	Topology
36	DRB1_0101 DRB1_0701 DRB1_1201 DRB1_1302 DRB1_1501	YDHIRIFYIKGREIK	**Antigen** **0.5444**	Allergen	Non-toxic	Inside
37	DRB1_0101 DRB1_0701 DRB1_1201 DRB1_1302 DRB1_1501	DHIRIFYIKGREIKT	**Non-antigen** **0.3165**	Allergen	Non-toxic	Inside
38	DRB1_0101 DRB1_0701 DRB1_1201 DRB1_1302 DRB1_1501	HIRIFYIKGREIKTS	**Antigen** **0.6721**	Allergen	Non-toxic	Inside
39	DRB1_0101 DRB1_0701 DRB1_1201 DRB1_1302 DRB1_1501	IRIFYIKGREIKTSL	**Antigen** **0.4284**	Allergen	Non-toxic	Inside
160	DRB1_0803 DRB1_1101 DRB1_1302 DRB1_1401	LAIGIYKVQRKEMEP	**Antigen** **1.3994**	Non-allergen	Non-toxic	Inside
161	DRB1_0803 DRB1_1101 DRB1_1302 DRB1_1401	AIGIYKVQRKEMEPK	**Antigen** **1.1907**	Non-allergen	Non-toxic	Inside
162	DRB1_0803 DRB1_1101 DRB1_1302 DRB1_1401	IGIYKVQRKEMEPKY	**Antigen** **1.1545**	Non-allergen	Non-toxic	Inside

### Protein-peptide docking analysis

After obtaining the 3D structure of HTL and CTL epitopes from the PEPFOLD server, PyRxVina was used to undertake a molecular docking research, utilizing 5 models of each epitope created by PEPFOLD 3. Seven T cell epitopes were docked with 4U6Y and 1BX2 receptors, and ten docked poses of each epitope were examined in PyMol using HLA alleles. The epitopes’ binding affinities revealed a strong interaction with their respective receptors (Table 5).

**Table 5:** Binding affinities of selected epitopes with HLA-A*01:01 and HLA-DRB1* 15:01

S. No	Receptor	Peptide (position)	Binding affinity (Kcal/mol)
1.	PDB ID:4U6Y	KVTVFNTNF(69)	−6.0
		IKSKIINPL(118)	−4.6
		KRSEWEVTL(55)	−5.3
		SEWEVTLNL(57)	−5.1
		LAIGIYKVQRKEMEP(160)	−4.8
		AIGIYKVQRKEMEPK(161)	−4.1
		IGIYKVQRKEMEPKY(162)	−5.0
2.	PDB ID:1BX2	KVTVFNTNF(69)	−6.1
		IKSKIINPL(118)	−5.2
		KRSEWEVTL(55)	−4.8
		SEWEVTLNL(57)	−6.1
		LAIGIYKVQRKEMEP(160)	−5.0
		AIGIYKVQRKEMEPK(161)	−4.2
		IGIYKVQRKEMEPKY(162)	−4.5

### Vaccine construction, properties prediction, and structural analysis

**Fig. 2:**
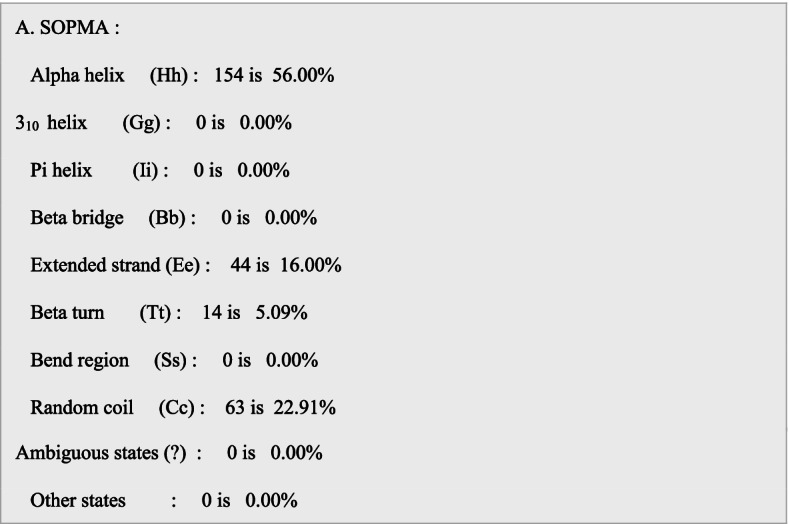
Secondary structure analysis by SOPMA

**Fig. 3:**
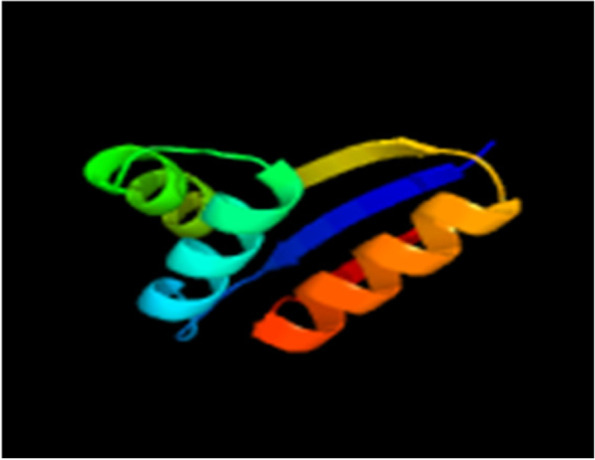
Predicted tertiary structure of vaccine in Phyre

**Fig. 4:**
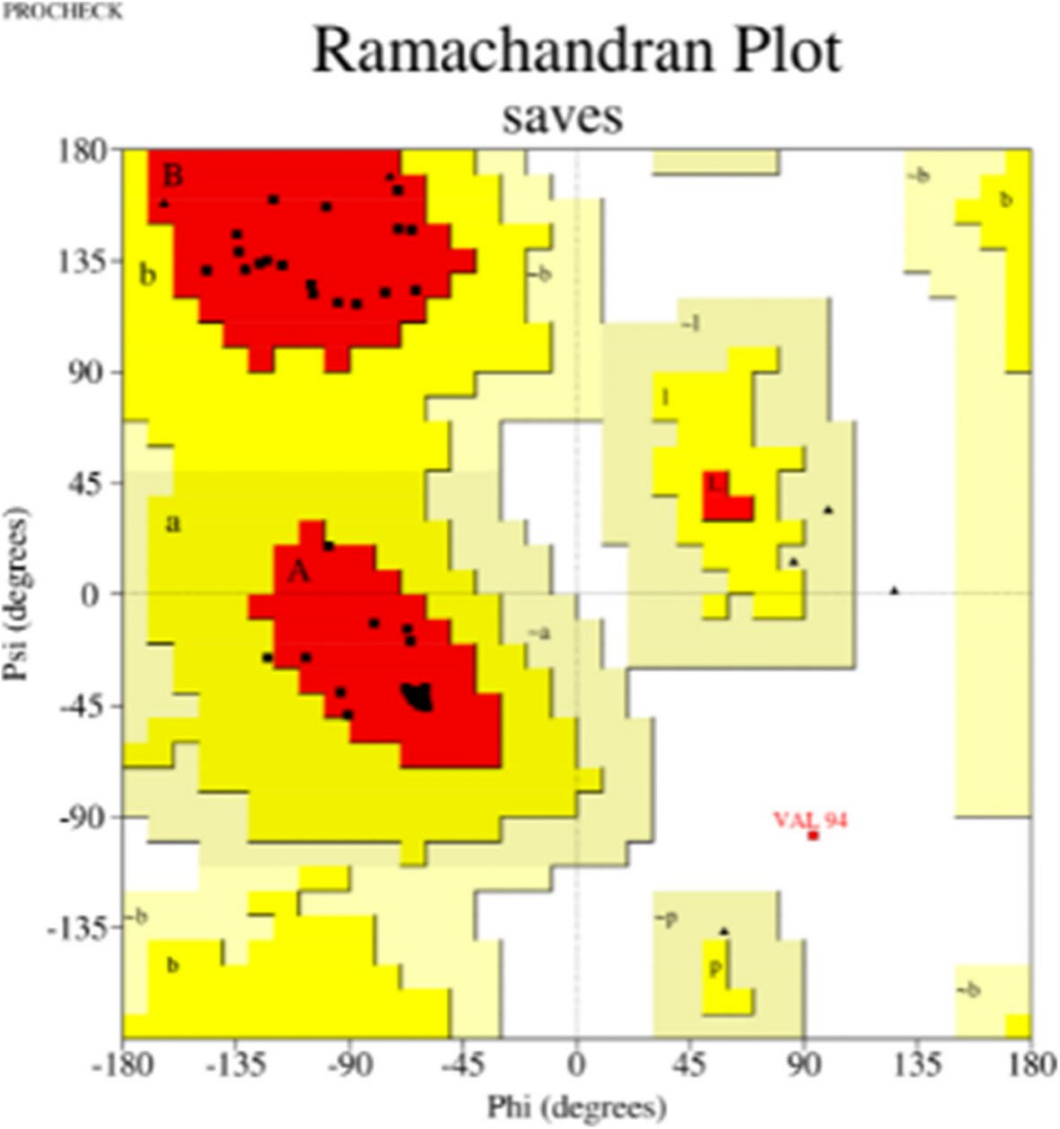
Validation of tertiary structure by Ramachandran plot analysis in PROCHECK server

**Fig. 5:**
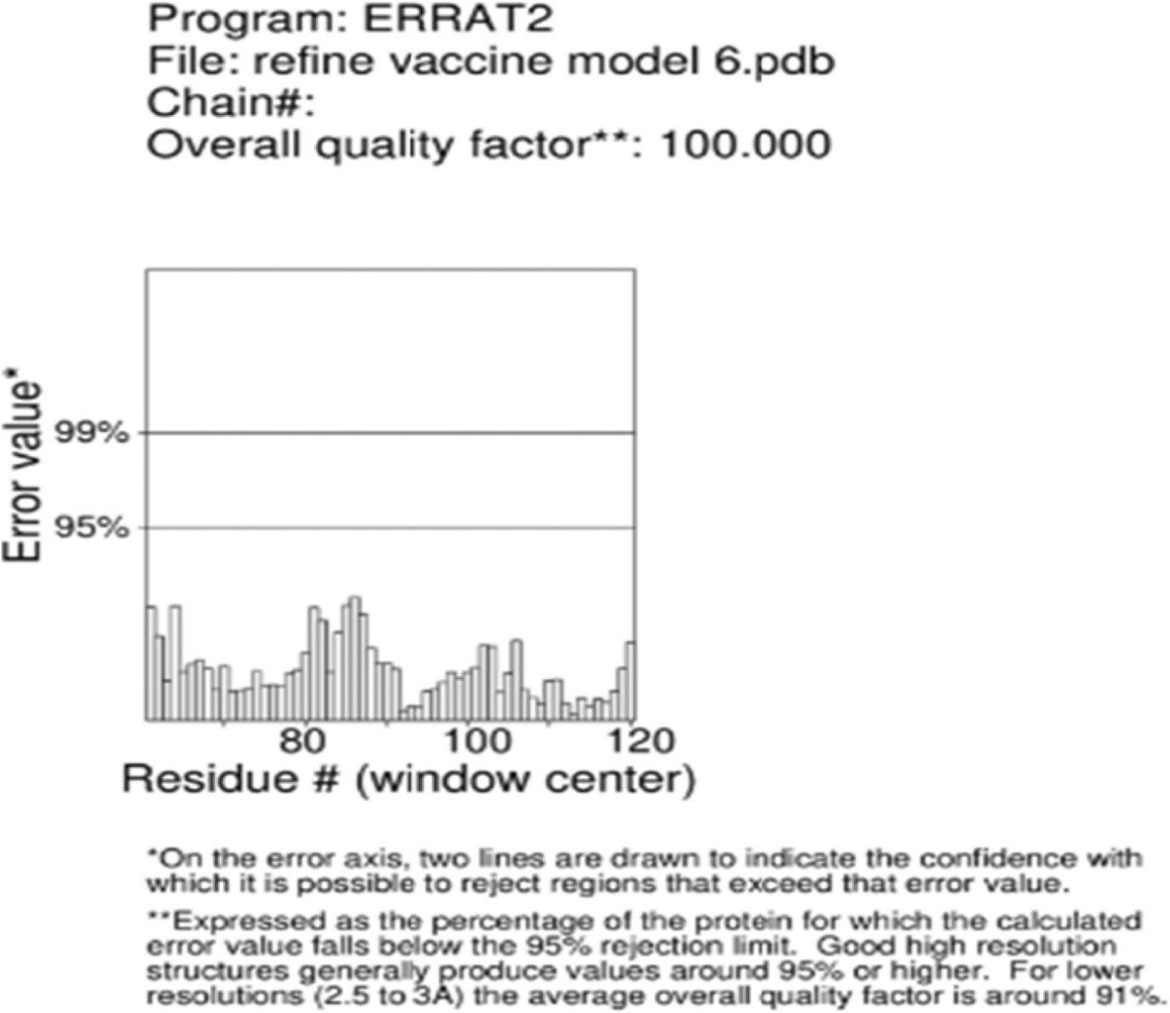
ERRAT RESULTS showing quality factor

**Fig. 6:**
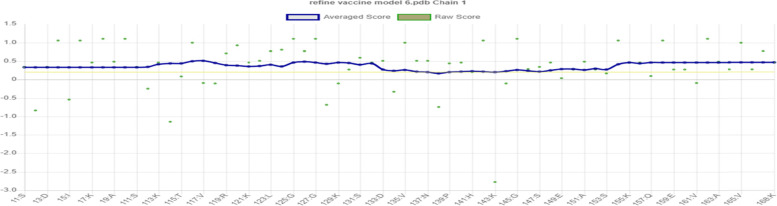
Represents VERIFY 3D results

L7/L12 ribosomal protein adjuvant is a 124 amino acid sequence used in vaccine development. The final vaccination sequence was 275 amino acids long, including 1 adjuvant, 4CTL, 3HTL, 2BCL epitopes, and numerous linkers. The proposed vaccine was projected to have an antigenicity of 0.7310, making it a possible antigen. The vaccination has been designed toward being non-allergic and non-toxic. The physicochemical parameters predicted by ProtParam were 30581.88 kDa molecular weight and 9.58 theoretical isoelectric point. Its half-life of mammalian reticulocytes, the instability index, overall aliphatic index, and the grand average of hydropathicity (GRAVY) were all expected to be 30 h, 19.41, 91.6, and −0.503, respectively. SOPMA’s secondary structure analysis revealed that it was made up of a 40.60% alpha helix, 24.14% extended stand, 13.73% extended strand, and 40.9% random coil (Fig. 2A). The vaccine’s tertiary structure, which was created in Phyre 2, was refined in Mod refiner saves ver. 6.0, which has 5 different parameter tools to evaluate the refined structure and the best model was chosen (Fig. 2B). Furthermore, PROCHECK’s Ramachandran plot demonstrated that 96.6% of residues were in the most desired areas, 1.7% in extra allowed regions, and 0% in liberally allowed regions (Fig. 2C). ERRAT (Fig. 2D). VERIFY 3D −98.53% of the residues have averaged 3D-1D score ≥0.2 - passed (Fig. 2E) Figs. 3, 4, 5 and 6.

### Molecular dynamics and protein-protein docking

**Fig. 7:**
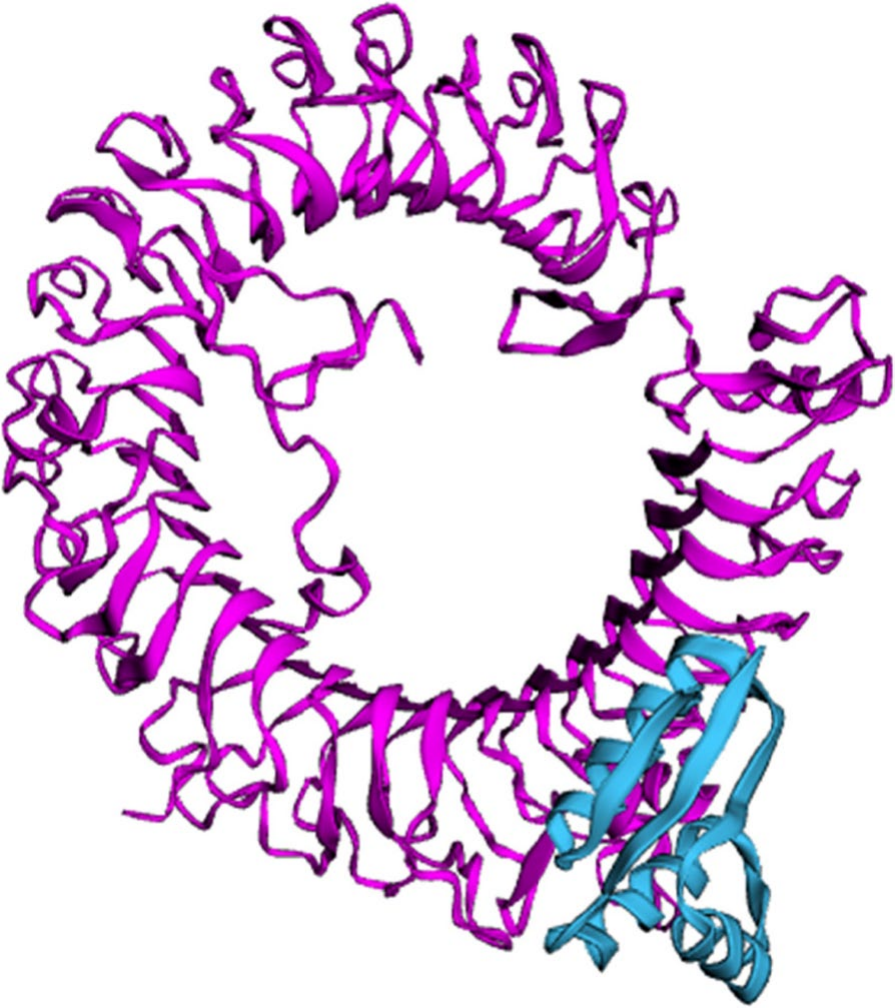
TLR-8 vaccine interaction predicted by HawkDock server. The vaccine is represented in blue and TLR-8 in pink

HawkDock server docked the vaccination with TLR-8 to elicit immunological responses using 10 models (Weng et al., 2019). We utilized a model with a docking score of −3935.49 and binding free energy of −18.28 Kcal/mol. TLR-8 and vaccine interacting residues were LEU (23), GLU (50), ILE (24), SER (53), GLN (57) and ARG (622), ASP (561), ILE (565), SRE (566), and TYR (536) as illustrated in Fig. 7 below.

**Fig. 8:**
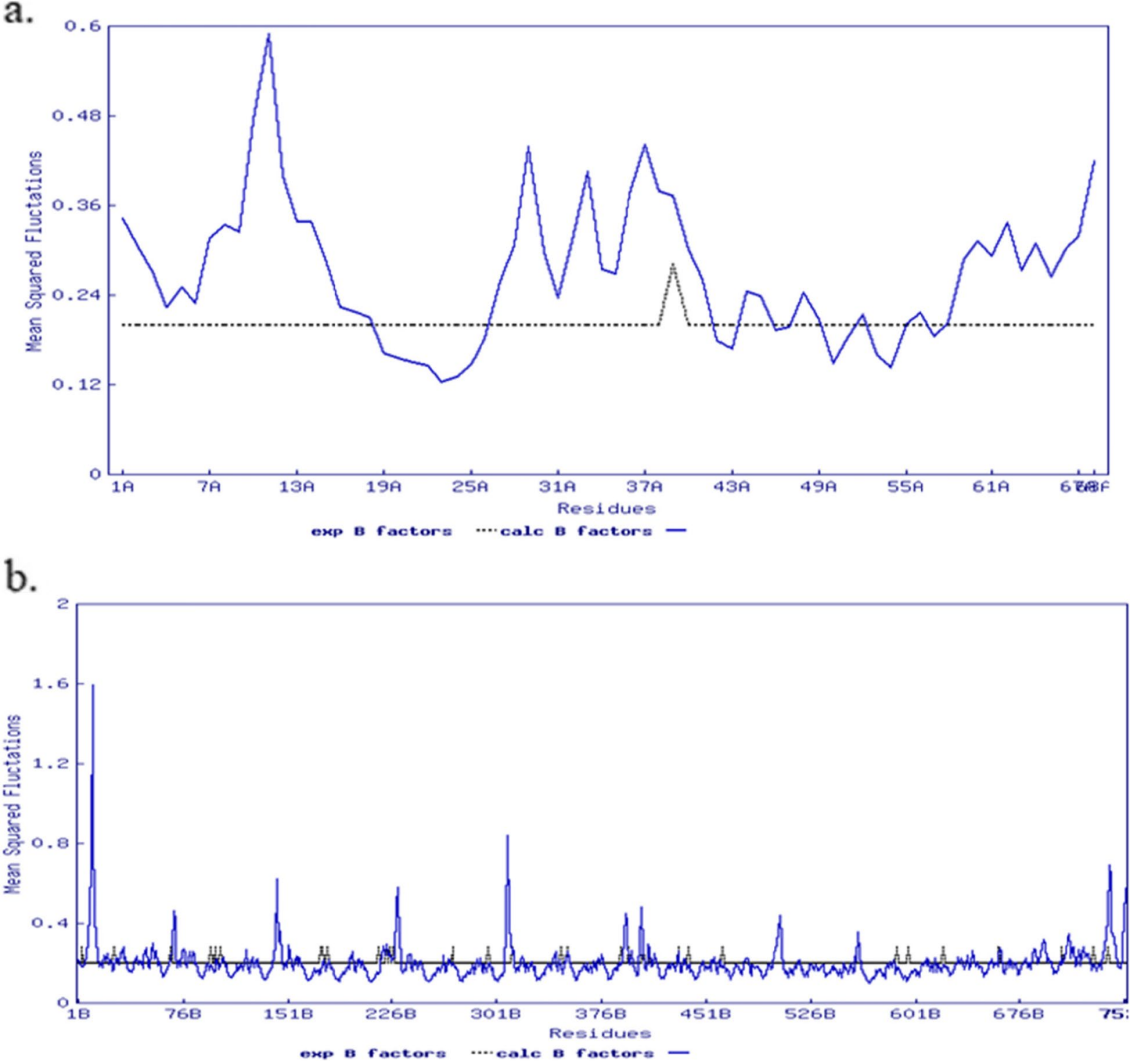
Molecular dynamics of docked complex. B-factor

**Fig. 9:**
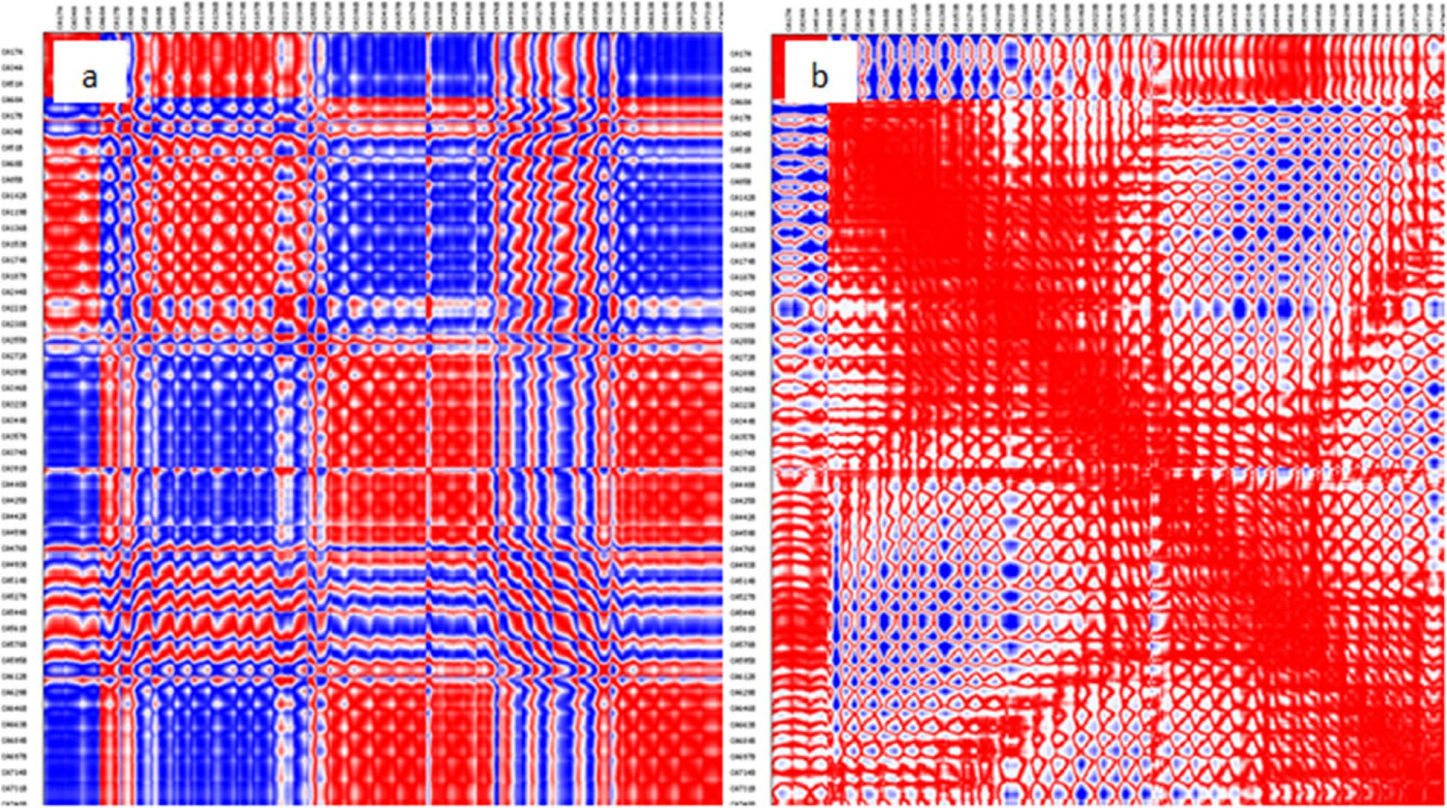
Molecular dynamics of docked complex. **a** Correlation maps. **b** Covariancce map

**Fig. 10:**
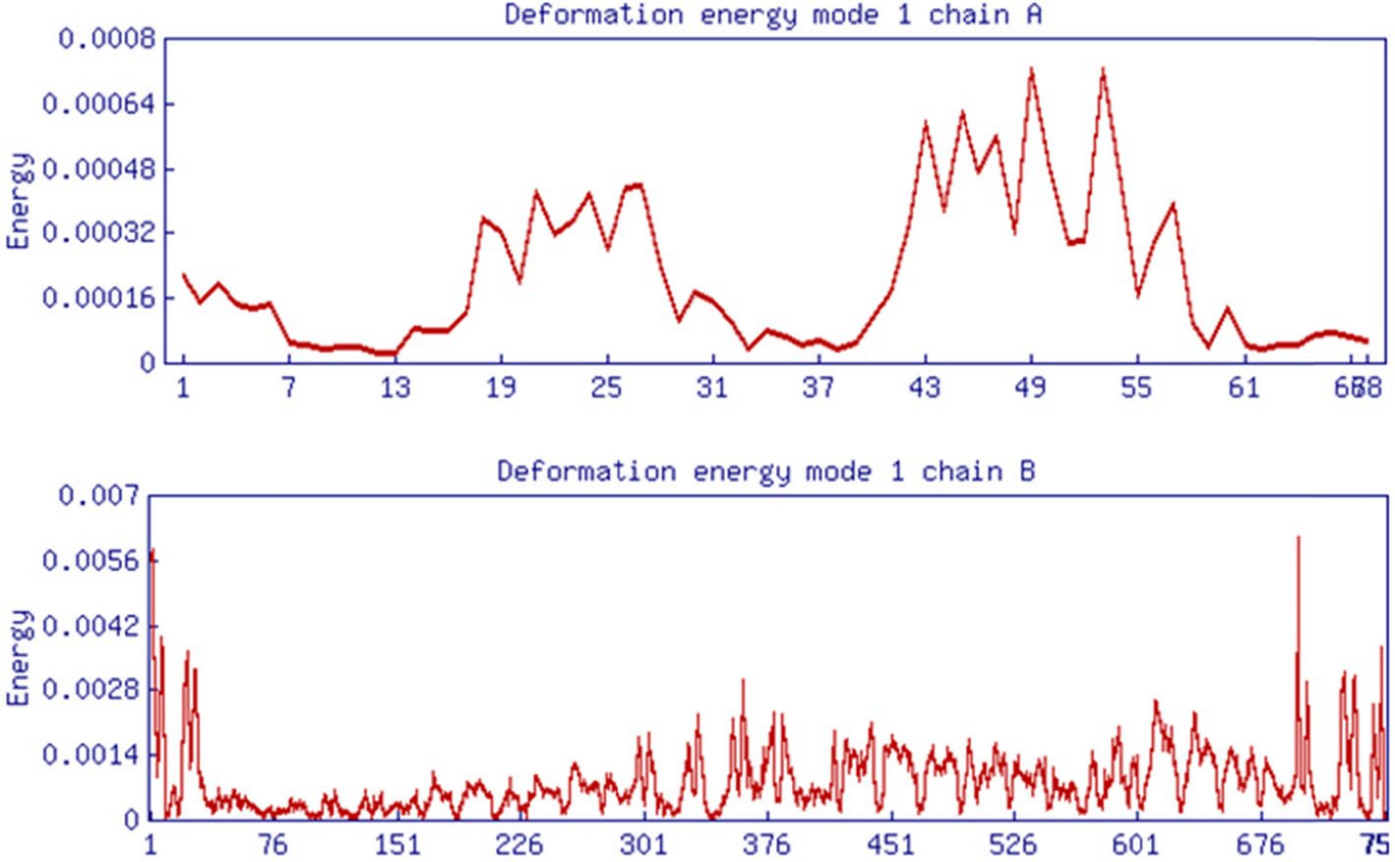
Molecular dynamics of docked complex. Deformation energies

**Fig. 11:**
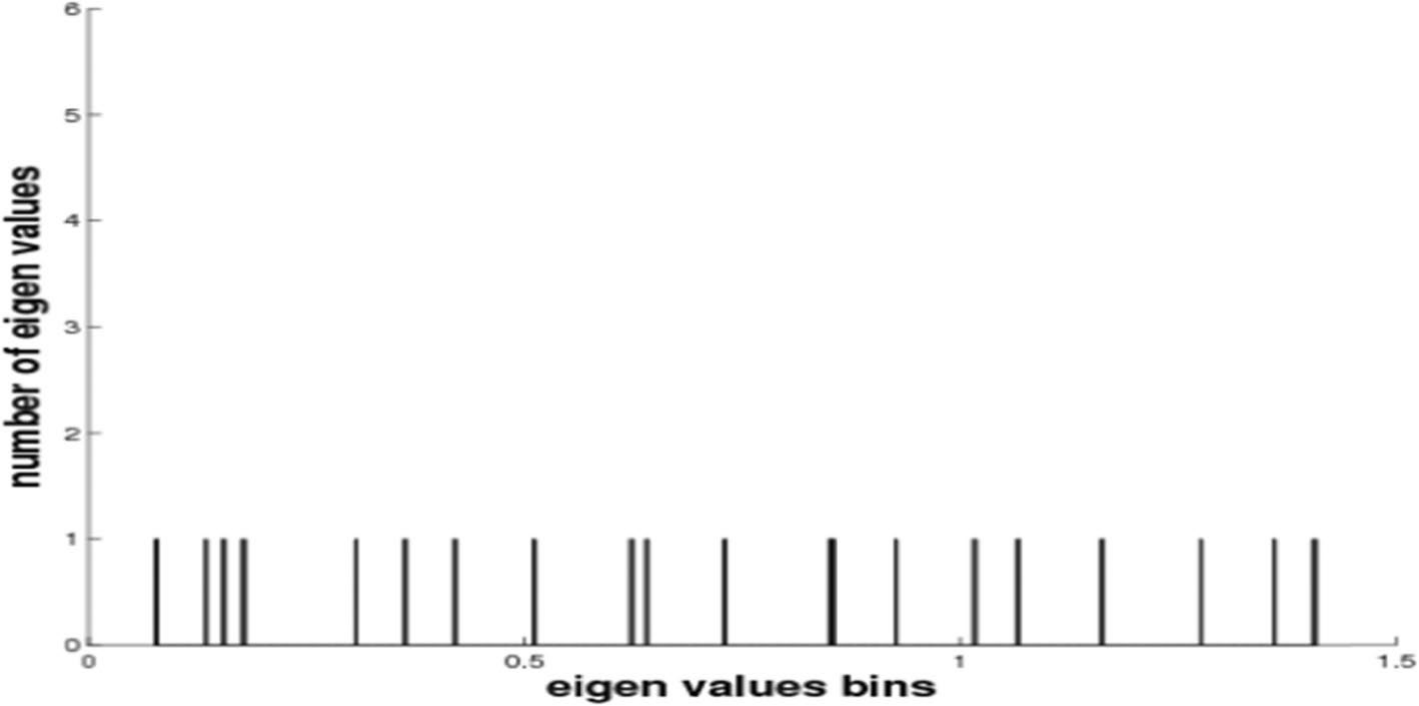
Molecular dynamics of the docked complex. Eigenvalues

Following that, molecular dynamic simulation studies on TLR-8 and vaccine docking were performed in the ANM 2.1 server. Peaks in (Fig. 8A) show B factor graphs of the receptor-ligand docked complex. Figure 9a, b shows the correlation map, whereas the covariance map shows the coupling between pairs of residues. The correlation is shown by red, non-correlation is indicated by white, and negative correlation is indicated by blue (Fig. 9). The deformation energies of both the chains were displayed in graphs (Fig. 10). Eigenvalues indicate the energy required to alter the structure: we discovered a TLR-8 and vaccine docked complex Eigenvalue 5.673205e−06 (Fig. 11).

### In silico cloning

**Fig. 12:**
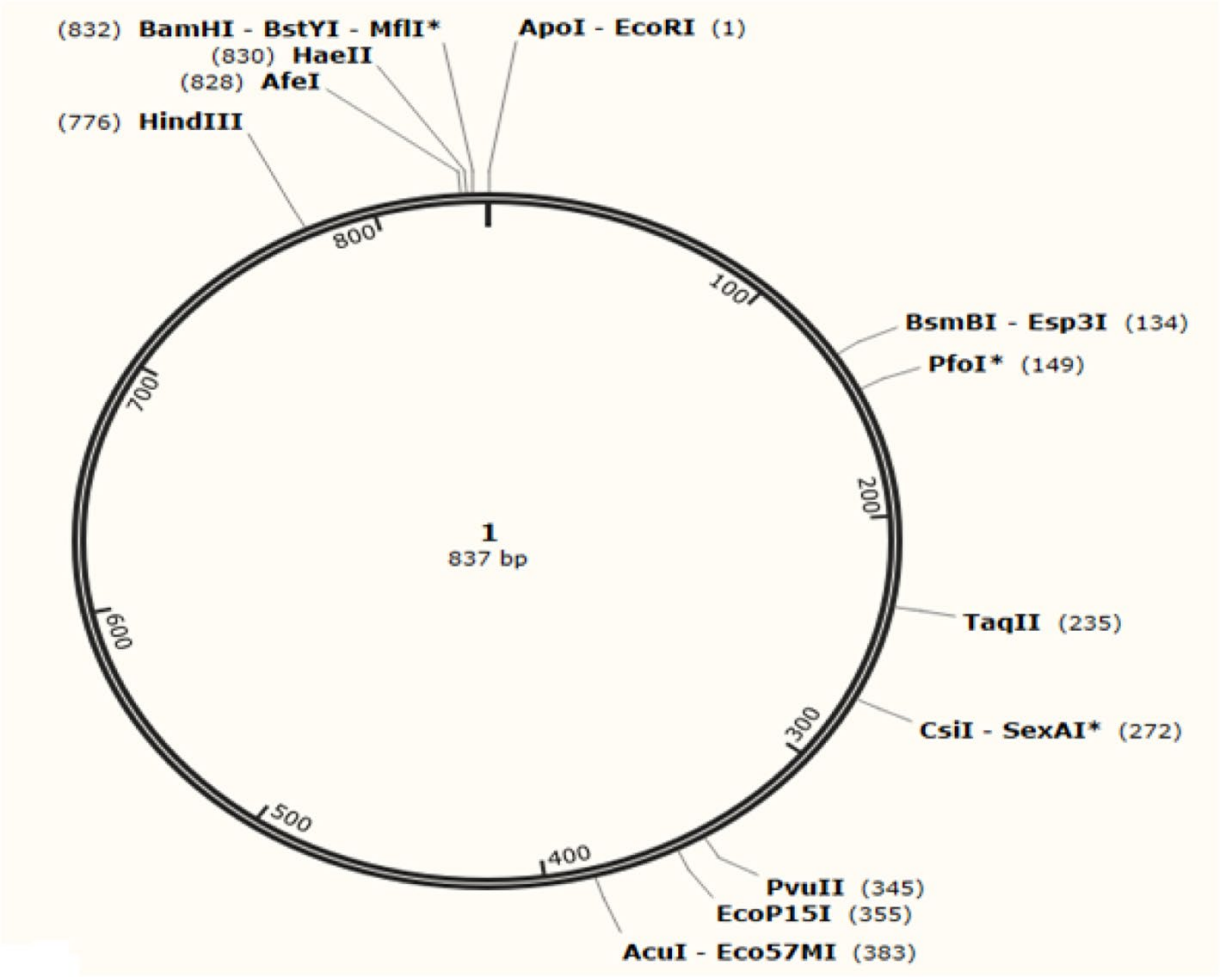
The in silico restricted cloning of the vaccine in plasmid vector PIB2 .5A. Shows the insertion of the EcoRI and BamHI in vector

**Fig. 13:**
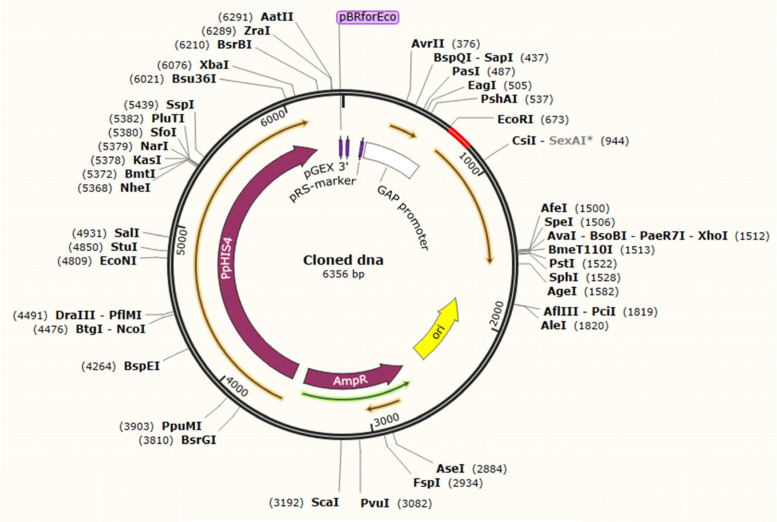
The cloned vaccine in vector plasmid

**Fig. 14:**
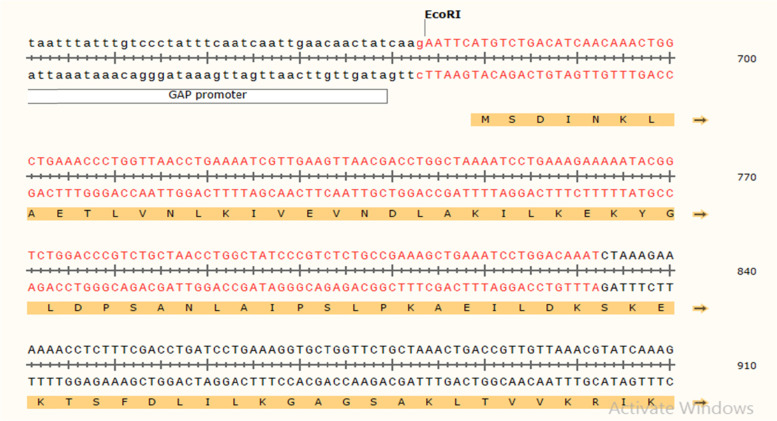
The sequence of the constructed vaccine in the plasmid PIB2

The Java Codon Adaptation Tool (JCat) optimized a codon sequence of 800 nucleotides with a codon adaptation index (CAI) of 0.99, effective vaccine expression in *E. coli*—K12 strain, and GC content of 45.2, resulting in favorable transcriptional translation efficiencies. The N- and C-terminal of EcoRI and BamHI restriction sites were connected using the SnapGene tool before introducing the codon sequence into the plasmid pIB2 vector, as shown in Figs. 12, 13, and 14). The plasmid was made up of 6356 base pairs after restriction cloning was used to introduce the optimal codon sequence.

## Discussion

The National Institute of Allergy and Infectious Diseases classifies bunyaviruses as a category, an emerging pathogen with the potential to cause considerable morbidity and death. (https://www.niaid.nih.gov/research/emerging-infectious-diseases-pathogens). This virus has no vaccination or antiviral treatment. Using several CTL, HTL, and B cell epitopes in a vaccine can stimulate both humoral and cellular immune responses with fewer side effects than a single epitope-based vaccine. Using an immunoinformatic method based on the virus nucleocapsid N-protein, we produced a multi-epitope vaccine for Bunyumwera virus. Several BCL and T cell epitopes have been discovered. After screening via a variety of immunological filters, just a few antigenic epitopes were carefully selected. As an adjuvant, L7/L12 ribosomal protein was used, as well as EAAAK, AAY, GPGPG, and KK for linking. The adjuvant stimulates TLR-4 and B cell inflammatory cytokine-induced innate immunity. The vaccine was cloned to enable expression and translation in a plasmid vector PIB2. It was expected to be antigenic, non-allergenic, and have a high binding affinity with TLR-8 in silico cloning. This multi-epitope vaccination may stimulate both innate and adaptive immunity.

## Conclusion

Computer modeling approaches help in the wide-scale screening of peptides with all potential HLA alleles to obtain the best peptides in a significant population. These approaches are effective in reducing the time and money spent on identifying high-specificity epitopes for vaccine design. The vaccine developed in this work was based on the nucleocapsid N-protein of the Bunyumwera virus and was created using a reverse vaccinology method. Further experimental validation is required to assess the vaccine’s therapeutic effectiveness and immunogenicity.

## Data Availability

Not applicable

## Abbreviations

HLA: Human leukocyte antigens
TLR-8: Toll-like receptor-8
GRAVY: Grand average of hydropathicity
NCBI: National Centre for Biotechnology Information
CTL: Cytotoxic T lymphocytes
DRB1: Protein-coding gene
HTL: Helper T lymphocytes
IEDB: Immune epitope database
MHC: Major histocompatibility complex
BCL: B cell lymphoma
SOPMA: Self-optimized prediction method with alignment
PDB: Protein Data Bank
